# Immunogenicity and safety of lyophilized and liquid dengue tetravalent vaccine candidate formulations in healthy adults: a randomized, phase 2 clinical trial

**DOI:** 10.1080/21645515.2020.1727697

**Published:** 2020-03-02

**Authors:** Mark Turner, Athanasia Papadimitriou, Peter Winkle, Nathan Segall, Michael Levin, Matthew Doust, Casey Johnson, Gregg Lucksinger, Carlos Fierro, Paul Pickrell, Marsha Raanan, Vianney Tricou, Astrid Borkowski, Derek Wallace

**Affiliations:** a Advanced Clinical Research, Meridian, ID, USA; b Takeda Pharmaceuticals International AG, Zurich, Switzerland; c Anaheim Clinical Trials, Anaheim, CA, USA; d Clinical Research Atlanta, Stockbridge, GA, USA; e Clinical Research Center of Nevada, Las Vegas, NV, USA; f Hope Research Institute, Phoenix, AZ, USA; g Johnson County Clin-Trials, Lenexa, KS, USA; h Tekton Research, Austin, TX, USA; i Takeda Vaccines Inc., Cambridge, MA, USA

**Keywords:** Dengue, vaccine, adults, tetravalent, Takeda

## Abstract

Takeda has developed a live-attenuated dengue tetravalent vaccine candidate (TAK-003) which has been shown to be immunogenic with acceptable reactogenicity in phase 1 trials. In agreement with World Health Organization prequalification requirements for dengue vaccines, Takeda has manufactured a lyophilized formulation of TAK-003 that allows stable storage at +2°C to +8°C. This randomized, double-blind, phase 2 study (NCT02193087) was performed in 1002 healthy dengue-naïve adults, 18–49 years of age, across seven centers in the USA to compare the safety and immunogenicity of one or two doses of a lyophilized TAK-003 formulation with the liquid TAK-003 formulation used in previous phase 1 studies. The primary objective was to show immunologic equivalence in terms of geometric mean titers (GMT) of neutralizing antibodies to the four dengue serotypes one month after one dose of the lyophilized and liquid formulations. Secondary assessments were of safety and seropositivity rates, including after a second dose. The primary endpoint was not met, because immunologic equivalence after one dose was only shown for the DENV-2 serotype. Nonetheless, GMTs and seropositivity rates to all four serotypes were achieved with all formulations after two doses and are in line with what was observed in previous studies. Additionally, in view of the acceptable reactogenicity, with no vaccine-related serious adverse events reported, these data support continuing further clinical development of the lyophilized TAK-003 formulation.

## Introduction

Dengue disease is caused by infection with one of the four dengue virus serotypes (DENV-1, DENV-2, DENV-3 and DENV-4) of the genus *Flavivirus*, family *Flaviviridae*.^1^ Dengue infections are responsible for increasing levels of illness in humans in an expanding range of countries, owing to the geographic spread of the mosquito vectors, *Aedes aegypti* and *Aedes albopictus*.^2^ A 2012 study found evidence of dengue viruses in 128 countries, with 3.9 billion people at risk of infection.^3^ The World Health Organization (WHO) believes there is substantial under-reporting of cases^4^ and recent estimates of the dengue burden indicate 50–100 million symptomatic cases per year representing 25% of all infections,^5,6^ and include about 20,000 deaths per year according to the WHO.^7^

While most dengue infections are asymptomatic, clinical cases can present from mild to severe disease, the most typical manifestation of disease being an influenza-like illness affecting all age groups. Symptoms include sudden onset of high fever variously accompanied by headache, retro-orbital pain, generalized myalgia and arthralgia, anorexia, abdominal pain and nausea.^5,7^ In a small number of severe dengue cases, dengue infection can result in severe intravascular leakage, multiple organ failure and death.^7^ There is no effective antiviral treatment to treat dengue/severe dengue, only supportive care. Infection with one dengue serotype will confer some immunity against that serotype, but not lasting heterologous cross-protection against other serotypes, and subsequent infection has been associated with more severe disease possibly because of immune enhancement of infection.^8^

The lack of effective antiviral treatment and increasing occurrence of dengue infections make development of effective vaccines a major medical requirement. Concerns about vaccine-induced immune enhancement of disease mean that dengue vaccines must be tetravalent, providing protective immunity against all four serotypes. Further, because severe dengue is a leading cause of serious illness and death among children in some Asian and Latin American countries,^9^ any vaccine should be available to include young children in this age range. However, the only currently licensed dengue vaccine is restricted to individuals aged 9–45 years who have evidence of previous exposure to dengue.^10^ Takeda’s candidate dengue vaccine (TAK-003) is a live tetravalent formulation based on genetically attenuated DENV-2 strain (TDV-2) with three chimeric viruses containing the DENV-1, DENV-3, and DENV-4 pre-membrane and envelope protein genes within the TDV-2 genetic backbone (TDV-1, TDV-3, and TDV-4).^11^ Phase 1 and 2 studies have demonstrated the safety, acceptable tolerability and immunogenicity of early TAK-003 formulations in healthy adults from endemic and non-endemic regions.^12–16^ The manufacturer’s decision to provide a liquid vaccine that can be lyophilized for storage in refrigerators at +2°–8°C is consistent with WHO prequalification which specifies a 6-months minimum supply storage capacity above +2ºC for new vaccines.^17^

The present study was conducted in healthy adults to assess the safety and immunogenicity of the new lyophilized formulation (manufactured at Lonza Houston Inc., and then mixed and lyophilized at IDT Biologika GmbH), with the primary objective of comparing equivalence of the immune responses after one dose of a lyophilized formulation and after one dose of the liquid formulation of TAK-003 (manufactured by Shantha Biotechnics Ltd) used in earlier phase 1 studies.

## Materials and methods

This was a randomized, double-blind phase 2 clinical trial conducted across seven centers in the United States from July 2014 to May 2015. The study protocol was approved by the IRB of each participating center and registered with ClinicalTrials.gov (NCT02193087). It was conducted according to current GCP and ICH guidelines. All participants provided written informed consent at enrollment. The primary objective was to compare the equivalence of liquid and lyophilized formulations of TAK-003 in terms of the geometric mean titers (GMT) of neutralizing antibodies elicited 30 days after vaccination. Secondary objectives included further comparative immunogenicity assessments of two doses of the lyophilized and liquid formulation vaccines at 120 days, and a comparative evaluation of the safety of the lyophilized and liquid formulations as measured by solicited local reactions and systemic adverse events (AEs) after each vaccination and by the occurrence of unsolicited AEs and Serious Adverse Events (SAEs).

### Participants

Eligible participants were adults of either gender, 18 to 49 years of age with no history of travel to a dengue endemic area within 6 months of study start. Inclusion criteria included: good health at the time of enrollment as determined by medical history and physical examination; BMI <35 kg/m^2^; and ability to comply with all study procedures for the duration of the trial. Main exclusion criteria included: any febrile illness at the time of enrollment; any history of chronic illness or therapy that might interfere with the trial results, including serological evidence of hepatitis B, hepatitis C, or HIV infection, and either documented or suspected flavivirus infection (including dengue, Japanese encephalitis, West Nile, yellow fever, or St. Louis encephalitis); receipt of other vaccinations within 14 days (for inactivated vaccines) or 28 days (for live vaccines) before enrollment or planned receipt of such vaccinations; and participation in any other clinical study. Sexually active females of childbearing potential had to have a negative pregnancy test at enrollment (repeated before each vaccination) and were required to practice an approved form of birth control for the duration of the study.

### Vaccine

TAK-003 consists of a molecularly characterized, attenuated DENV-2 strain (TDV-2) and DENV-2/1 (TDV-1), DENV-2/3 (TDV-3), and DENV-2/4 (TDV-4) chimeras expressing the pre-membrane (prM) and envelope (E) surface antigens corresponding to dengue serotypes 1, 3 and 4, respectively, genetically inserted into the DENV-2 backbone. Two vaccine formulations were used, one presented in both liquid and lyophilized formulations, each dose containing identical dosages in plaque-forming units (pfu) of the four dengue vaccine strains: 2 × 10^4^ pfu TDV-1; 5 × 10^4^ pfu TDV-2; 1 × 10^5^ pfu TDV-3; 3 × 10^5^ pfu TDV-4. The Shantha liquid vaccine formulation previously established to be safe and immunogenic in phase 1 studies^12–14^ was stored at −60°C or below. Prior to administration, Shantha vaccine was thawed at room temperature and diluted 1:5 in vaccine diluent. The other liquid vaccine formulation (IDT liquid) was stored at −60°C or below, and the lyophilized formulation (IDT lyophilized) at 2°–8°C in a refrigerator. Before administration, the IDT lyophilized formulation was reconstituted by adding 0.7 mL of water for injection.

### Study design

Enrolled participants were randomly assigned (2:1:1:6) to one of four study groups (with intended numbers of participants) to receive the following vaccinations: Group A (n = 200) one dose of Shantha liquid; Group B (n = 100) two doses of Shantha liquid; Group C (n = 100) two doses of IDT liquid; Group D (n = 600) two doses of IDT lyophilized. Vaccines were administered by subcutaneous injection in the upper arm on Day 1 and Day 90, with Group A receiving placebo (phosphate-buffered saline) on Day 90.

### Safety assessment

All participants were monitored for 30 minutes after each vaccination for immediate reactions, and participants were instructed to record on diary cards solicited local reactions (pain, erythema and swelling) for 7 days, solicited systemic adverse events (AE) (headache, malaise, myalgia and asthenia) and oral temperature daily for 14 days, and any unsolicited AEs up to 28 days post-vaccination. Serious AEs (SAEs) were reported throughout the study duration. Further safety analyses included assessment of vital signs and clinical laboratory parameters. Blood samples were drawn from subsets of participants at Days 1, 8, 15, 90, 97 and 104 for clinical laboratory safety analyses. All solicited AEs were graded 1–3 for severity, grade 1 (mild) being easily tolerated, grade 2 (moderate) interfered with normal activity, grade 3 (severe) prevented normal activity. Erythema and swelling were graded 1 (2.5–5 cm diameter), 2 (5.1–10 cm) and 3 (>10 cm), and oral temperatures as 1 (38.0º–38.4ºC), 2 (38.5º–38.9ºC), and 3 (39.0º–40.0ºC). The investigator assessed solicited systemic AEs, unsolicited AEs and SAEs for relatedness; relatedness for SAEs was also determined by the sponsor.

### Immunogenicity assessment

Blood samples were drawn from all participants on Days 1, 30, 90 and 120 and shipped to Focus Diagnostics Inc. (San Juan Capistrano, CA, USA) for immunogenicity analyses by micro-plaque reduction neutralization test (PRNT),^18^ in this report termed a microneutralization test (MNT). Analyses measured dengue virus plaque reduction neutralization antibodies expressed as the titer resulting in 50% plaque reduction.

### Statistics

This study was designed to assess the safety and immunogenicity of vaccination with two liquid (Shantha and IDT liquid) and one lyophilized (IDT lyophilized) formulations of TAK-003 in healthy adults. The primary comparison was to test equivalence of the immunogenicity at Day 30 of IDT lyophilized formulation (Group D) and the Shantha liquid formulation (Groups A + B combined), as determined by a margin of 0.67 to 1.5 in the ratio of GMTs of neutralizing antibodies between the two formulations for all four serotypes. Assuming a one-sided significance level of 0.05 based on the TOST (Two One-Sided Tests) procedure,^19^ a true ratio of GMT for the 2 formulations to be 1, and SDs in the natural logarithm of titers for 4 serotypes distributed as N (1.5, SD = 0.2), a sample size of 900 (300/600), adjusted for approximately 5% dropout, was sufficient to achieve 95% power for showing equivalence of the two formulations in Groups A + B combined vs. Group D for one serotype, and greater than 80% power for showing equivalence of the two formulations for all four serotypes. The distribution of SDs in the natural logarithm of titers for 4 serotypes was estimated from previous TAK-003 studies.^12–16^ The power calculations were based on 5,000 Monte Carlo simulation runs, using R. The IDT liquid formulation (Group C) was included to determine whether there was a manufacturing effect in addition to a formulation effect. Immunogenicity comparisons between the 2 liquid vaccines (Groups A + B combined vs Group C) or between IDT liquid and IDT lyophilized formulations (Group C vs Group D) were exploratory. As a result, a total sample size of 1,000 was planned for the study, with a ratio of 2:1:1:6 for groups A, B, C and D.

## Results

**Figure 1.**
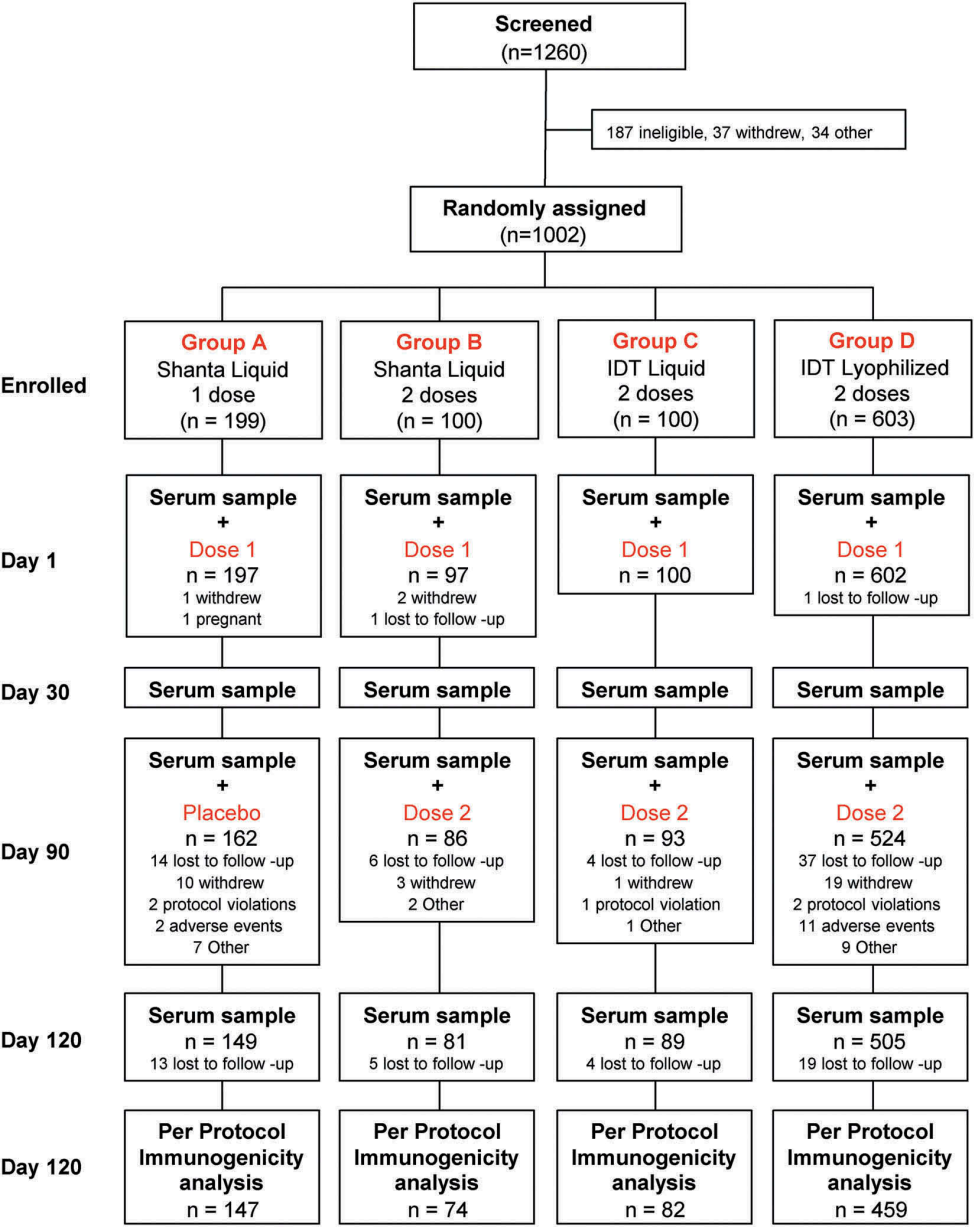
Study design and subject disposition

In total, 1260 18–49-year-old adult volunteers of either gender were screened and 1002 met the study entry criteria, and were enrolled and randomly assigned into the four study groups in a 2:1:1:6 ratio. Of these, 996 vaccinated participants were included in the Safety Set for analysis as six did not receive any vaccination: one had a positive pregnancy test at screening and five did not return or withdrew prior to vaccination (Figure 1). Of these 996, 131 participants did not receive a second study vaccination (61 were lost to follow-up, 33 withdrew, 13 had adverse events (AEs) leading to discontinuation and 24 for other reasons). One subject was withdrawn after an unrelated serious adverse event (SAE). A further 102 participants were seropositive for at least one dengue serotype on Day 1 and were excluded from the Per Protocol set. This led to a total of 762 (76.5%) vaccinated participants who completed the study according to protocol and were included in the Per Protocol immunogenicity analyses. The demographic characteristics of the Per Protocol set show similar distributions across groups in terms of age, weight, gender and race (Table 1).

**Table 1.** Demographics of the Per Protocol study population in the immunogenicity analysis

	Group A Shantha Liquid 1 Dose (n = 147)	Group B Shantha Liquid 2 Doses (n = 74)	Group C IDT Liquid 2 Doses (n = 82)	Group D IDT Lyophilized 2 Doses (n = 459)
**Age**, years (Mean ± SD)	32.2 ± 8.91	31.5 ± 8.81	34.3 ± 9.05	32.1 ± 8.88
**Male** (%)	46.3	44.6	51.2	49.0
**Weight**, kg (Mean ± SD)	77.9 ± 14.7	79.2 ± 17.2	77.2 ± 15.7	78.2 ± 15.9
**BMI**, kg/m^2^ (Mean ± SD)	26.8 ± 4.13	27.1 ± 4.55	26.7 ± 4.94	26.8 ± 4.58
**Race** (%)				
Asian	1.4	0	2.4	2.6
African American	26.5	31.1	26.8	24.8
Caucasian	64.6	64.9	69.5	66.9
Other	7.5	4.1	1.2	5.7

SD, standard deviation; BMI, body mass index.

### Immunogenicity

#### After one dose

**Figure 2.**
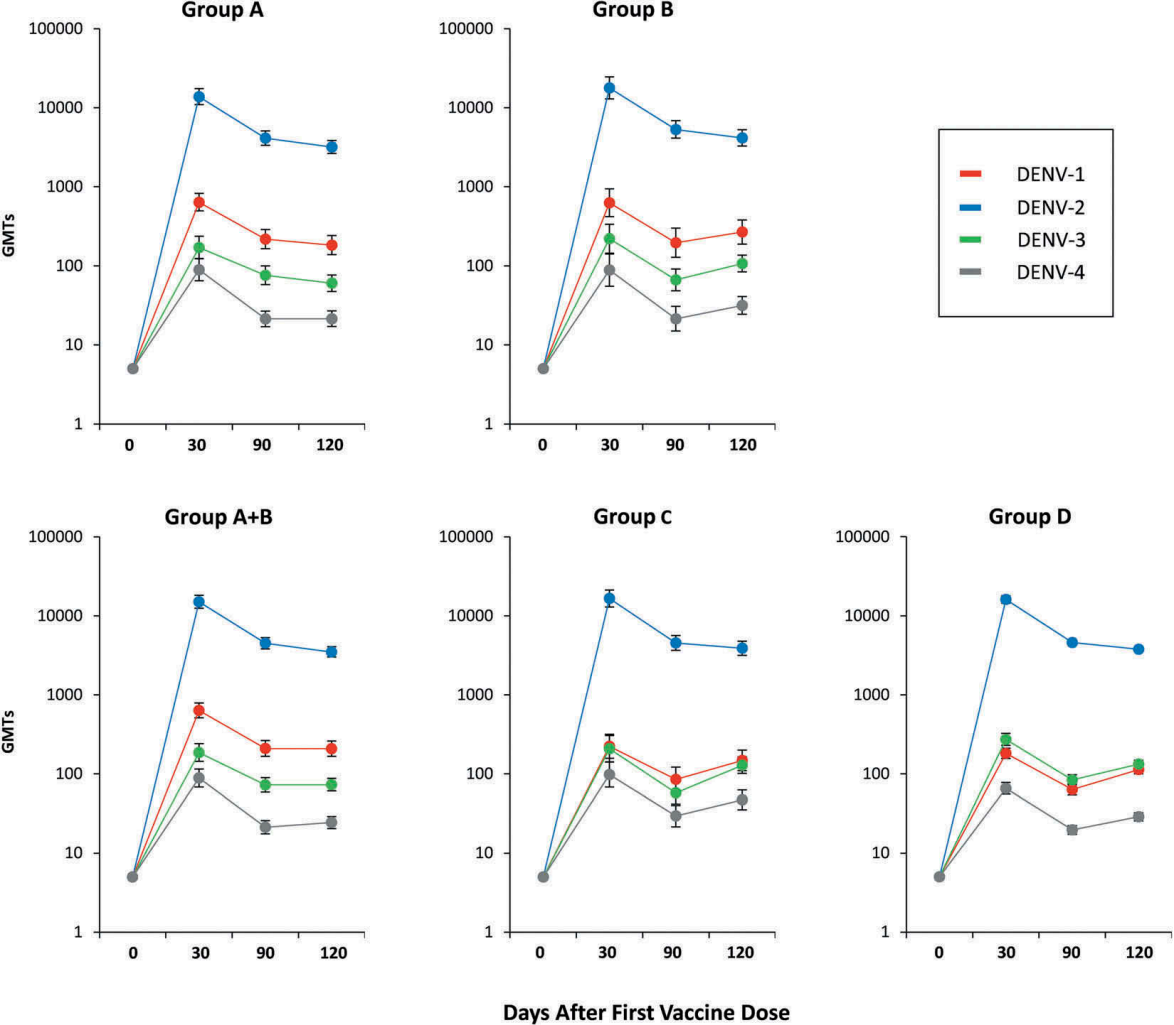
Geometric Mean Titers (GMTs) of serotype-specific antibodies (with 90% CI bars) at baseline and 30, 90, and 120 days after administration of the first vaccine dose (Per Protocol set)

**Figure 3.**
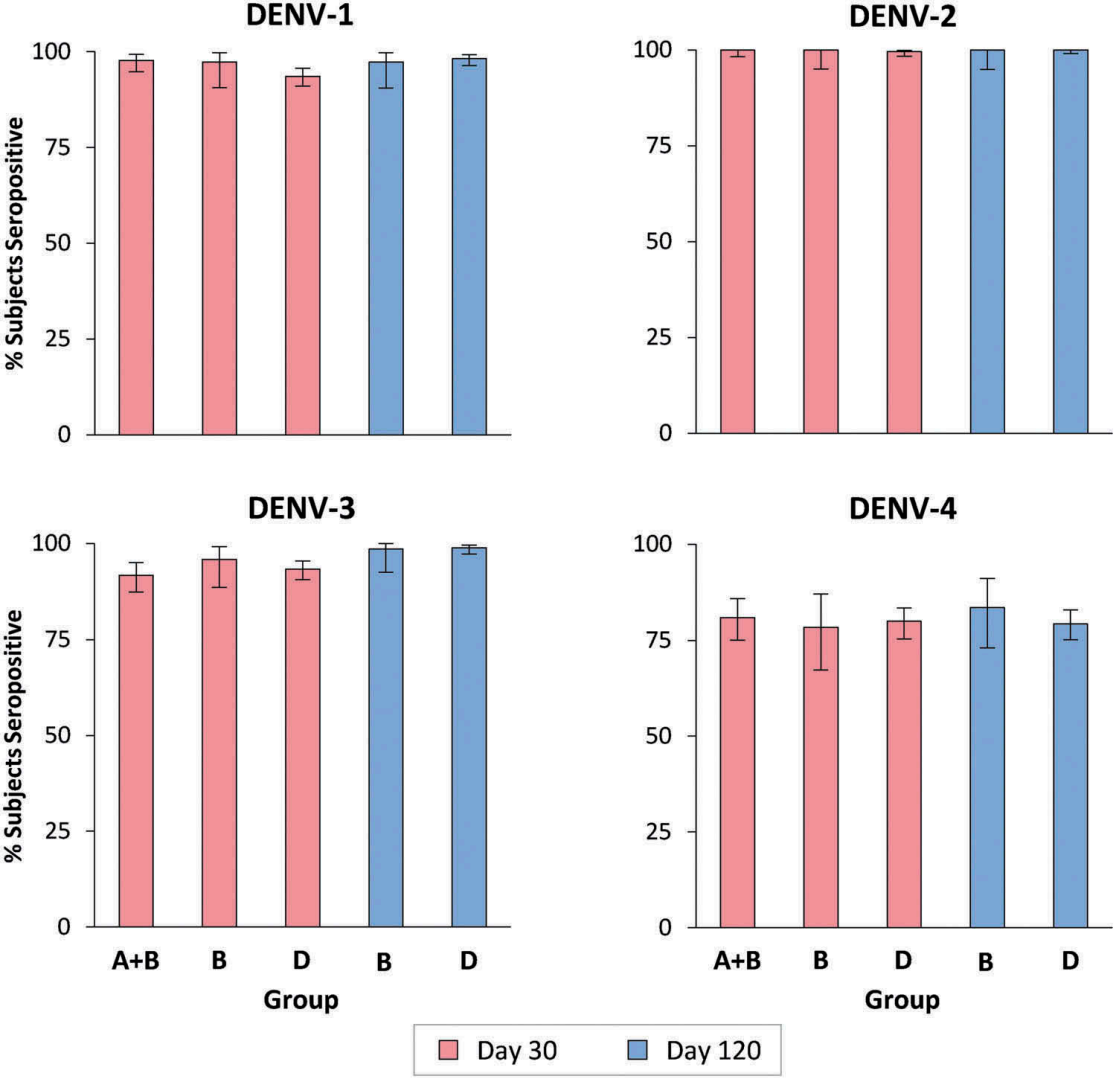
Serotype-specific seropositivity rates (with 90% CI bars) 30 days after administration of first (Day 30) and second (Day 120) vaccine doses (Per Protocol set)

Neutralizing antibody responses for each of the four DENV serotypes, measured as plaque reduction neutralization titers (PRNT) resulting in 50% reduction in plaques (MNT_50_) and expressed in geometric mean titers (GMT) are shown in Figure 2 for the 4 study groups. One dose of either formulation induced robust antibody responses against all four serotypes 30 days later. In magnitude, the highest antibody responses were to DENV-2 (at Day 30, DENV-2 GMTs were 13,868 to 17,877 depending on the study groups), followed by DENV-1 (181 to 639), then DENV-3 (171 to 273) and DENV-4 (66 to 98). At this time-point, after one dose, DENV-2, DENV-3 and DENV-4 GMTs were similar for all study groups. However, there was a noticeable difference in DENV-1 GMTs between study groups, with lower GMTs in Groups C and D after the IDT liquid and lyophilized formulations than the groups who received the Shantha liquid formulation (Groups A and B). The primary endpoint of immunological equivalence (defined as the 90% CIs for GMT ratios being contained within the boundaries of 0.67–1.50) was demonstrated between IDT lyophilized (Group D) and Shantha liquid (Group A + B) formulations on Day 30 for DENV-2, although not for DENV-1, DENV-3, and DENV-4. The differences in magnitude of antibody responses across serotypes did not result in any statistically significant differences in seropositivity rates (which were defined as percentages of subjects with reciprocal neutralizing titers ≥10 for each of the DENV serotypes) between groups (Figure 3), which were high for both Shantha liquid (Groups A + B) and IDT lyophilized (Group D) TAK-003: DENV-1, 97.7% & 93.6%; DENV-2, 100% & 99.6%; DENV-3, 91.8% & 93.4%; DENV-4, 80.9% & 80.0%. Overall, despite the lower GMTs observed against DENV-1 30 days after Dose 1, the lyophilized IDT formulation induced similar seropositivity rates to the Shantha liquid formulation.

#### After two doses

In the two groups who received a second dose of Shantha liquid (Group B) or IDT lyophilized (Group D) at Day 90, the serotype-specific antibody levels 30 days later (Day 120) were slightly lower than those observed at Day 30 (Figure 2). Serotype-specific GMTs in the two groups were similar except for DENV-1, for which the GMT was higher after the Shantha liquid vaccine than the IDT lyophilized formulation. At this time-point equivalence was demonstrated between the Shantha liquid (Group B) and IDT lyophilized (Group D) TAK-003 formulations for DENV-2 and DENV-4, but not for DENV-1 and DENV-3. As noted at Day 30, high levels of seropositivity were observed at Day 120 in Groups B and D (Figure 3): DENV-1, 97.3% & 98.2%; DENV-2, 100% & 100%; DENV-3, 98.6% & 98.9%; DENV-4, 83.6% & 79.3%, with no statistically significant differences between the two. Although only included for an exploratory analysis of immunogenicity, antibody responses in Group C after one or two doses of the IDT vaccine as a liquid formulation were similar to the responses in Group D after the IDT lyophilized formulation for all serotypes at all time-points measured as GMTs or seropositivity rates.

### Safety

Overall the vaccinations were well tolerated with few severe adverse events (AEs) and no vaccine-related serious AEs (SAEs) occurred during the study. One SAE led to withdrawal of a Group D participant from the trial following a complex partial seizure after the first dose, though it was not considered related to the vaccination. The subject was withdrawn and did not receive the second dose.

The frequency of SAEs was low in all groups and there was no notable difference in the percentages of subjects who experienced SAEs in the liquid (1.0%; Group B) and lyophilized (Group D; 1.3%) TAK-003 groups. Severe AEs were reported by less than 5% of each study group. The frequency of vaccine-related non-serious AEs was similar in the liquid (15.5%; Group B – Shantha liquid) and IDT lyophilized (18.1%; Group D) vaccine groups (Table 2). Likewise, the percentages of subjects who experienced any unsolicited AEs were similar in the two-dose liquid (47.4%; Group B – Shantha liquid) and IDT lyophilized (43.7%; Group D) TAK-003 groups.

**Table 2.** Percentages of subjects experiencing unsolicited adverse events within 28 days of administration of first and second vaccine doses (safety analysis set)

	Subjects with adverse events (%)				
	Group A Shantha Liquid 1 Dose (n = 197)	Group B Shantha Liquid 2 Doses (n = 97)	Group A + B Shantha Liquid 1 or 2 Doses (n = 294)	Group C IDT Liquid 2 Doses (n = 100)	Group D IDT Lyophilized 2 Doses (n = 602)
Any AE	40.1	47.4	42.5	45.0	43.7
Vaccine-related AE	18.8	15.5	17.7	22.0	18.1
Severe AE	2.5	4.1	3.1	2.0	3.5
SAE	1.0	1.0	1.0	0	1.3
Vaccine-related SAE	0	0	0	0	0
AE Leading to Withdrawal	0	0	0	0	0.2
SAE Leading to Withdrawal	0	0	0	0	0.2

AE, adverse event; SAE, serious adverse event.

#### Local reactions

**Figure 4.**
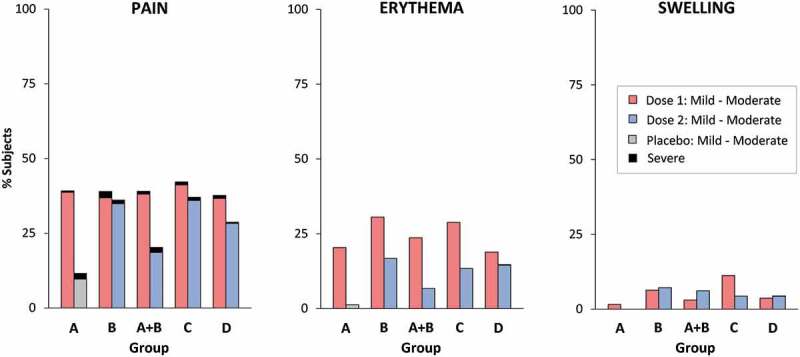
Percentages of subjects experiencing solicited local reactions within 7 days of vaccination (safety analysis set). Severe pain defined as significant pain at rest, or pain preventing normal activity. Severe erythema or swelling defined as >10 cm

Generally, all TAK-003 formulations were well tolerated with few severe solicited local reactions in the 7 days post-vaccination (Figure 4). The most frequent local reaction was mild to moderate injection site pain, followed by erythema and swelling. Incidences of these reactions were similar for the first doses of both liquid and lyophilized formulations, and there was a trend for rates to be lower after the second dose, particularly for Shantha liquid TAK-003 There were some cases of severe pain, which were more frequent with first and second doses of Shantha liquid than with the IDT lyophilized TAK-003. There were few cases of severe erythema or swelling.

#### Systemic adverse events

Solicited systemic adverse events were reported at similar rates in all vaccine groups, for both liquid and lyophilized formulations, in the 14 days after the first and second doses (Table 3). Most of these events were described as mild to moderate, with less than 4% of subjects reporting severe systemic AEs. There were no reports of severe fever (oral temperature ≥39.0ºC) in any participant in any group. The most frequent systemic AE was headache, occurring after approximately one third of the vaccinations. Severe headache was more frequent after the first dose of Shantha liquid TAK-003 (Group A + B, 3.8%) than after the IDT lyophilized (Group D, 1.9%), but the event occurred in 2.9% of subjects after the second dose of both vaccine formulations. Proportionally fewer subjects experienced severe myalgia after their second vaccination with the IDT lyophilized formulation (0.4%; Group D) than with the Shantha liquid formulation (2.4%; Group B).

**Table 3.** Percentages of subjects experiencing mild to moderate (and severe) solicited systemic adverse events within 14 days of vaccination (safety analysis set)

	Percentages of subjects in each group with solicited systemic adverse events (severe in parentheses)									
	Group A Shantha Liquid 1 Dose (n = 197)		Group B Shantha Liquid 2 Doses (n = 97)		Group A + B Shantha Liquid 1 or 2 Doses (n = 294)		Group C IDT Liquid 2 Doses (n = 100)		Group D IDT Lyophilized 2 Doses (n = 602)	
	Dose 1	Placebo	Dose 1	Dose 2	Dose 1	Dose 2	Dose 1	Dose 2	Dose 1	Dose 2
Headache	31.4 (3.1)	16.8 (3.2)	30.5 (5.3)	21.7 (2.4)	31.1 (3.8)	18.5 (2.9)	29.6 (1.0)	18.0 (3.4)	32.8 (1.9)	20.0 (2.9)
Asthenia	20.4 (2.1)	9.7 (1.3)	20.0 (0)	10.8 (1.2)	20.3. (1.4)	10.1 (1.3)	18.4 (1.0)	13.5 (1.1)	19.6 (2.1)	10.8 (0.6)
Malaise	21.5 (3.7)	12.9 (1.3)	7.4 (0)	10.8 (1.2)	21.3 (2.8)	12.2 (1.3)	16.3 (1.0)	13.5 (2.2)	19.1 (1.7)	11.8 (1.0)
Myalgia	23.0 (1.0)	14.2 (1.3)	31.6 (2.1)	18.1 (2.4)	25.9 (1.4)	15.5 (1.7)	22.4 (2.0)	18.0 (0)	25.8 (1.0)	15.7 (0.4)
Fever	1.6 (0)	0 (0)	1.1 (0)	2.4 (0)	1.4 (0)	0.8 (0)	1.0 (0)	0 (0)	1.4 (0)	0.4 (0)

Severe fever described as body temperature >39.0ºC. Severe headache, asthenia, malaise, and myalgia all described as that preventing normal activity.

Participants in Group C, who were administered with the IDT liquid for exploratory purposes, displayed a similar solicited reactogenicity profile to the IDT lyophilized formulation administered to Group D, with no meaningful differences in incidences or severity of local reactions or systemic AEs. There were no clinically important changes in vital signs. Overall 6.7% of subjects had markedly abnormal values for vital signs parameters reported during the study, but with no clinically important differences between groups. There were no clinically important changes in hematology and chemistry test results from baseline at any of the scheduled visits. Overall, markedly abnormal hematology test results and chemistry results were reported for 7.8% and 3.7% of subjects, respectively, but with no clinically meaningful differences between groups.

## Discussion

This study was designed to compare the immunogenicity and safety profiles of a lyophilized formulation of TAK-003 with that of the Shantha formulation previously used in phase 1 and 2 clinical trials.^12–16^ The IDT formulation met the newly introduced WHO prequalification requirement for storage above +2°C.^17^ The study failed to meet the primary endpoint of immunological equivalence of the lyophilized and liquid formulations, which could only be demonstrated for one serotype (DENV-2) after one dose, and for two serotypes (DENV-2 and DENV-4) after two doses, when assessed as GMTs of serotype-specific neutralizing antibodies. Although immunological equivalence in terms of GMTs for all four serotypes between the lyophilized and liquid formulations could not be shown, we do not consider this as being relevant enough to prevent further clinical development of IDT lyophilized formulation. All formulations induced similar seropositivity rates with no statistically significant differences after one or two doses, indicating a vaccine effect regardless of formulation.

Overall, the lyophilized and liquid vaccine formulations were generally well tolerated. No vaccine-related SAEs were reported and reactogenicity profiles of all formulations were consistent with those in previous reports of TAK-003.^12–16,20–24^

The reasons for the differences in the magnitude of the immune responses between lyophilized and liquid formulations are unknown, but the immunological equivalence achieved for DENV-2 and DENV-4, as well as the limited differences in GMTs between IDT liquid and lyophilized formulations, suggest that the failure of equivalence for DENV-1 and DENV-3 is not related to the lyophilization process. Indeed, the elicited increases in GMTs and high levels of seropositivity to all serotypes achieved in this trial supported the further development of TAK-003 as a lyophilized vaccine. Following the initiation of this study, a decision was made to use in all future clinical trials a lyophilized formulation with TDV-2 potency reduced by one log relative to the other serotypes, in order to promote a more balanced immune response to all four serotypes. Recent data from a phase 2 trial assessing the immunogenicity and safety of a single dose of either lyophilized formulation indicated a more balanced immune response with the new lyophilized formulation, particularly in subjects who were seronegative prior to vaccination.^20^ Also, another phase 2 clinical trial conducted in Asia and Latin America has demonstrated this lyophilized formulation with reduced TDV-2 potency to be highly immunogenic.^21,22^ That study has reported high immunogenicity and acceptable reactogenicity in subjects from dengue-endemic countries, notably in children from 2 to 8 years of age for whom there is no vaccine currently available. Eighteen-month interim results from that trial also indicate that TAK-003-vaccinees have a significantly lower risk of dengue infection (relative risk 0.29 (95% CI 0.13–0.72) than control vaccine recipients.^22^ Recently, in an on-going phase 3 pivotal trial (ClinialTrials.gov NCT02747927) TAK-003 met the trial primary efficacy endpoint by showing prevention of dengue fever in ~20,100 4–16-year-old children living in dengue-endemic countries through 12 months after a 2-dose regimen.^23^

A limitation of the present study is that no formal comparison was made with the new IDT formulation in liquid form, although a study arm (Group C) administered with this vaccine was included for exploratory comparisons. On the other hand, the consistency of the safety and immunological data from this group compared with the others does not indicate that any formal differences would have been found to explain the failure to meet immunologic equivalence. Of note, no abnormalities were observed regarding the stability of either formulation. Only humoral immunogenicity was assessed in this trial, but TAK-003 is known to stimulate cell-mediated immune responses to dengue non-structural proteins,^24^ which may make a significant contribution to the protection against dengue disease.^25^ This aspect of the immune response was not tested with the lyophilized TAK-003 in this study but is being investigated in other ongoing clinical studies. It should be noted that although this report terms antibody responses as ‘serotype-specific’, the possibility of cross-reactivity between serotypes should not be discounted.

### Conclusions

The Shantha liquid TAK-003 formulation has previously been shown to be safe, generally well tolerated and immunogenic for all four serotypes. Although immunologic equivalence of the new IDT lyophilized formulation with the Shantha vaccine could only be shown for serotype 2 after the first dose (primary endpoint based on antibody GMTs), and for serotypes 2 and 4 after two doses, there was no meaningful difference in the overall immunogenicity of the two formulations. Both liquid and lyophilized formulations were well tolerated with similar overall safety profiles. Little or no manufacturing effect can be concluded given that no consistent differences were seen between the liquid and lyophilized TAK-003 formulations with regard to immune responses and the overall safety and reactogenicity profiles, thereby fulfilling the WHO prequalification requirement on storage conditions for new vaccines. Results of this study support the further clinical development of the IDT lyophilized formulation which is already being used in ongoing clinical studies to determine its clinical effectiveness against dengue disease.
